# Genome Sequence of the Saprophyte *Leptospira biflexa* Provides Insights into the Evolution of *Leptospira* and the Pathogenesis of Leptospirosis

**DOI:** 10.1371/journal.pone.0001607

**Published:** 2008-02-13

**Authors:** Mathieu Picardeau, Dieter M. Bulach, Christiane Bouchier, Richard L. Zuerner, Nora Zidane, Peter J. Wilson, Sophie Creno, Elizabeth S. Kuczek, Simona Bommezzadri, John C. Davis, Annette McGrath, Matthew J. Johnson, Caroline Boursaux-Eude, Torsten Seemann, Zoé Rouy, Ross L. Coppel, Julian I. Rood, Aurélie Lajus, John K. Davies, Claudine Médigue, Ben Adler

**Affiliations:** 1 Unité de Biologie des Spirochètes, Institut Pasteur, Paris, France; 2 Victorian Bioinformatics Consortium, Monash University, Clayton, Victoria, Australia; 3 Australian Research Council Centre of Excellence in Structural and Functional Microbial Genomics, Department of Microbiology, Monash University, Clayton, Victoria, Australia; 4 Australian Bacterial Pathogenesis Program, Department of Microbiology, Monash University, Clayton, Victoria, Australia; 5 Plate-forme Génomique, Institut Pasteur, Paris, France; 6 Bacterial Diseases of Livestock Research Unit, National Animal Disease Center (NADC), Agricultural Research Service (ARS), United States Department of Agriculture (USDA), Ames, Iowa, United States of America; 7 Australian Genome Research Facility, Gehrmann Laboratories, University of Queensland, St. Lucia, Queensland, Australia; 8 Plate-forme Intégration et Analyse génomique, Institut Pasteur, Paris, France; 9 Commissariat à l'Energie Atomique (CEA), Direction des Sciences du Vivant, Laboratoire de Génomique Comparative, Institut de Génomique, Genoscope, Evry, France; 10 Centre National de la Recherche Scientifique (CNRS) UMR8030, Génomique Métabolique, Evry, France; University of Minnesota, United States of America

## Abstract

*Leptospira biflexa* is a free-living saprophytic spirochete present in aquatic environments. We determined the genome sequence of *L. biflexa*, making it the first saprophytic *Leptospira* to be sequenced. The *L. biflexa* genome has 3,590 protein-coding genes distributed across three circular replicons: the major 3,604 chromosome, a smaller 278-kb replicon that also carries essential genes, and a third 74-kb replicon. Comparative sequence analysis provides evidence that *L. biflexa* is an excellent model for the study of *Leptospira* evolution; we conclude that 2052 genes (61%) represent a progenitor genome that existed before divergence of pathogenic and saprophytic *Leptospira* species. Comparisons of the *L. biflexa* genome with two pathogenic *Leptospira* species reveal several major findings. Nearly one-third of the *L. biflexa* genes are absent in pathogenic *Leptospira*. We suggest that once incorporated into the *L. biflexa* genome, laterally transferred DNA undergoes minimal rearrangement due to physical restrictions imposed by high gene density and limited presence of transposable elements. In contrast, the genomes of pathogenic *Leptospira* species undergo frequent rearrangements, often involving recombination between insertion sequences. Identification of genes common to the two pathogenic species, *L. borgpetersenii* and *L. interrogans*, but absent in *L. biflexa*, is consistent with a role for these genes in pathogenesis. Differences in environmental sensing capacities of *L. biflexa, L. borgpetersenii,* and *L. interrogans* suggest a model which postulates that loss of signal transduction functions in *L. borgpetersenii* has impaired its survival outside a mammalian host, whereas *L. interrogans* has retained environmental sensory functions that facilitate disease transmission through water.

## Introduction

The genus *Leptospira* contains pathogenic and saprophytic species that differ in their capacity for survival in a vast array of environments that range from soil and water [1] to the tissues of mammalian hosts during acute and chronic infection [2]. Typically, long term colonization by pathogenic *Leptospira* of the proximal renal tubules of mammalian maintenance host species provides a persistent source of infection; thus, pathogenic *Leptospira* is shed in the urine of chronically infected animals, facilitating transmission to naïve hosts [2]. Leptospirosis is of considerable importance to international public health, with more than half a million cases reported annually due largely to environmental exposure to pathogenic *Leptospira* species, with mortality rates of up to 25% in some outbreaks. In addition, leptospirosis in production animals results in a significant economic burden worldwide [2].

Recent applications of molecular taxonomy techniques to this genus reveal extensive genetic diversity within *Leptospira*, with more than 16 pathogenic and saprophytic species recognized [3], [4]. A significant challenge in the future will be to more precisely correlate these genetic differences with biological differences. The relationship between leptospiral genome content, pathogenesis and the ability to survive in diverse environmental niches is a particularly important area of investigation, highlighted by our recent findings. A process of genome erosion and loss of gene function in *L. borgpetersenii* serovar Hardjo [5] limits its viability outside a mammalian host and likely impairs disease transmission through water, a route commonly used by *L. interrogans* to infect new hosts.

To gain insight into the genetic potential of *Leptospira* and to help identify genes that contribute to long-term survival in surface water, we determined the genome sequences of two *L. biflexa* strains, the first saprophytic *Leptospira* species to be characterized by genome analysis. Comparison of these data to genomes of the pathogenic species *L. borgpetersenii* [5], and *L. interrogans* [6], [7] provides an opportunity to identify features that are unique to pathogenic and saprophytic species, thereby providing new experimental directions and novel perspectives on leptospiral evolution, environmental persistence and the causation of disease.

## Results and Discussion

### *Leptospira* genomes vary in replicon content and genetic organization

**Figure 1:**
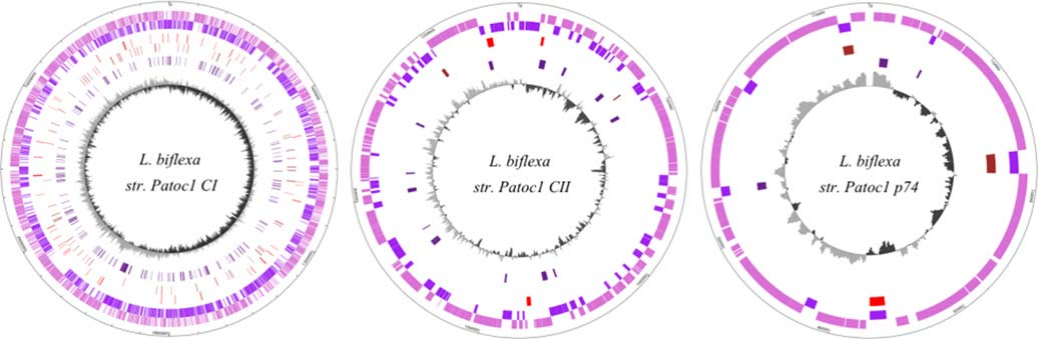
Circular maps of the three *L. biflexa* replicons. (1) the coordinates in bp beginning at 0 = oriC; (2) dark pink: genes unique to *L. biflexa*, not found in *L. interrogans* serovar Copenhageni and *L. borgpetersenii* serovar Hardjobovis (identity >40% over 80% of the length of the smallest protein). (3) dark purple: genes found in *L. biflexa*, *L. interrogans* and *L. borgpetersenii* (identity >40% over 80% of the length of the smallest protein). (4) red: genes found in *L. biflexa* and *L. borgpetersenii*, but not in *L. interrogans* (identity >40% over 80% of the length of the smallest protein). (5) brown : genes found in *L. biflexa* and *L. interrogans*, but not in *L. borgpetersenii* (identity >40% over 80% of the length of the smallest protein). (6) blue: genes found in *L. biflexa* and other sequenced spirochetes (*Borrelia afzelii* PKo, *Borrelia burgdorferi*, *Borrelia garinii*, *Treponema denticola* and *Treponema pallidum*) (identity >40% over 80% of the length of the smallest protein ). (7) The innermost ring shows GC skew; positive skew is shown in grey, and negative skew is shown in black.

The genome of *Leptospira biflexa* serovar Patoc strain Patoc1 (Ames strain) consists of three replicons with a total of 3,956,086 base pairs (bp) (Figure 1). The two larger replicons share extensive similarity to the two chromosomes that comprise the genomes of *L. borgpetersenii* and *L. interrogans* and are therefore referred to as chromosome I (CI; 3,603,977 bp; GC% 38.89) and chromosome II (CII; 277,995 bp; GC% 39.27). *L. biflexa* possesses a third circular replicon (74,114 bp; GC% 37.47), that we designate p74, not found in the previously sequenced pathogenic *Leptospira* species. (The complete genomic sequences of *L. biflexa* serovar Patoc strain Patoc1, strains Paris and Ames, have been deposited in GenBank under the Accession Numbers: CP000777, CP000778, CP000779, CP000786, CP000787 and CP000788).

A total of 3,590 protein-coding genes (CDSs) was identified in *L. biflexa* (Table 1). Most of these genes are located on CI, including two *rrf* genes, two *rrl* genes and two *rrs* genes, coding for 5S, 23S and 16S rRNA molecules respectively. These rRNA genes are not linked to each other, a feature unusual among most bacteria but common among *Leptospira*. Similar to the slower-growing pathogenic species *L. interrogans* and *L. borgpetersenii,* each of which has a relatively low number (37) of transfer RNA (tRNA) genes, the faster-growing *L. biflexa* surprisingly has only 35 tRNA genes. This finding indicates that the growth rate of *Leptospira* is not restricted by the low number of tRNA and rRNA genes as previously suggested [7], but rather may be due to other differing metabolic capacities amongst *Leptospira* spp. As reported for other *Leptospira* spp. [8], essential genes such as *gltB* (glutamate synthase) and *asd* (aspartate semialdehyde dehydrogenase) are carried on CII in *L. biflexa*.

**Table 1:** Summary of genome features of pathogenic and saprophytic *Leptospira*

Features	¥ *L. borgpetersenii*		¥ *L. interrogans*		¥ *L. biflexa*			
	CI	CII	CI	CII	CI	CII	P74	LE-1 prophage^b^
Size (bp)	3,614,456	317,335	4,277,185	350,181	3,603,977	277,995	74,116	73,623
G+C content (%)	41.0	41.2	35.1	35.0	38.9	39.3	37.5	38.5
Protein-coding percentage	80	80	74.9	75.5	92.3	93.3	90.9	93.4
**Protein coding sequences**								
CDS^a^	2,607	237	3,105	274	3,268	266	56	82
With assigned function	1,644	135	1,817	159	2,042	141	31	19
Conserved hypothetical	373	32	484	34	464	43	5	2
Unique hypothetical	590	70	804	81	762	82	20	61
**Transposases**	215	26	26	0	8	1	1	0
**Pseudogenes**	340	28	38	3	32	1	0	0
**Transfer RNA genes**	37	0	37	0	35	0	0	0
**Ribosomal RNA genes**								
23S	2	0	2	0	2	0	0	0
16S	2	0	2	0	2	0	0	0
5S	1	0	1	0	2	0	0	0

¥ *L. borgpetersenii* serovar Hardjo strain L550, *L. interrogans* serovar Copenhageni strain Fiocruz, *L. biflexa* serovar Patoc strain Ames

a excluding transposases and pseudogenes

b [11], [38]

It is unclear if replicon III (p74) functions as a chromosome, i.e. carries genes essential for survival, or is an extrachromosomal element or plasmid. Thirteen genes on p74 have orthologs located on CI in pathogenic *Leptospira* (Table 2). For example, *recBCD* are located on CI in *L. interrogans* and *L. borgpetersenii,* but are located on p74 in *L. biflexa.* Mutation of these housekeeping genes in other bacterial species can affect transformation competence and viability [9], suggesting that p74 is essential for the survival of *L. biflexa*.

**Table 2:** CDS from Replicon III (p74) that have an ortholog in Chromosome I in other *Leptospira*

Stop	start	locus_tag	ortholog_tag	product
4267	3794	LBF_5005	SPN2759	Conserved hypothetical protein
5304	4993	LBF_5007	SPN2285	Conserved hypothetical protein
7485	5608	LBF_5009	SPN2142	Serine phosphatase RsbU, regulator of sigma subunit
10908	11909	LBF_5013	SPN2858	ABC-type Fe3+-siderophore transport system, permease component
16746	18548	LBF_5018	SPN2289	Exodeoxyribonuclease V, alpha subunit
18545	22168	LBF_5019	SPN2290	Exodeoxyribonuclease V, beta subunit
22171	25470	LBF_5020	SPN2291	Exodeoxyribonuclease V, gamma subunit
41270	42064	LBF_5030	SPN0228	Bacteriophage-related protein*
53226	52999	LBF_5037	SPN1718	Conserved hypothetical protein
60761	60456	LBF_5044	SPN3221	Antitoxin of toxin-antitoxin stability system
61204	60767	LBF_5045	SPN3222	Hypothetical protein
62047	63093	LBF_5047	SPN1129	Homoserine kinase
63112	64008	LBF_5048	SPN1151	GGDEF domain receiver component of a two-component response regulator

* Ortholog found on Chromosome II or Chromosome I in other *Leptospira*.

GC skew analysis (Figure 1) suggests that CI, CII and p74 are theta-type replicons that replicate bi-directionally from a unique origin. Each *L. biflexa* replicon encodes its own partition proteins from origin-proximal genes that may recognize and interact with a replication-specific binding site. The replication origin of CI resembles typical circular chromosomes from other bacteria, i.e. it is adjacent to *dnaA*, which encodes the initiator protein, and other genes such as *dnaN*, *recF* and *gyrAB*. In contrast, the replication origins of CII, replicon p74 and the previously reported leptophage LE1 [10] resemble phage and plasmid replicons; they contain both a partition operon and a downstream putative *rep* gene. For p74, the predicted replication origin based on GC skew analysis includes a partition operon (pLEPBI0001/LBF_5000 and pLEPBI0002/LBF_5001) and an adjacent gene, pLEPBI0003/LBF_5003, the product of which shares 63% similarity with the leptophage LE1 Rep protein. Regardless of the similarity between the LE1 Rep protein and the product of pLEPBI003, p74 and the LE1 prophage can co-exist (data not shown). Furthermore, we demonstrated that this region of p74 directs autonomous replication in *L. biflexa* (Figure S1). This will facilitate the construction of new shuttle vectors for the genetics of *Leptospira* and their use in co-transformation experiments with the *L. biflexa*-*E. coli* shuttle vector derived from LE1 [11].

We suggest that the CII and p74 replicons evolved from a stabilized circular intermediate of a progenitor phage related to LE1 [10]. Intermediates in this process may be related to the LaiGI-1 Genomic Island [12], which exists both as an element integrated into CI of *L. interrogans* serovar Lai or as an autonomously replicating plasmid. Stabilization of these intermediates could have occurred through illegitimate recombination resulting in incorporation of essential genes from CI into the smaller replicons. Evidence for this model is provided by the presence of genes located on the CI replicon in pathogenic *Leptospira* on either CII or p74 in *L. biflexa*, as noted above.

The genus *Leptospira* is renowned for the stability of its agglutinating antigens during *in vitro* culture, with examples of strains maintaining serovar identity during more than 80 years of propagation [2], implying considerable genomic stability in the absence of selective pressure for antigenic change. Furthermore, more than 2% of the *L. interrogans* serovar Copenhageni genome is dedicated to LPS biosynthesis. In *L. biflexa* about 1.4% of the genome encodes LPS biosynthesis functions with essentially all relevant CDS encoded on the same strand, and these genes are identical in the Ames and Paris strains. In contrast, non-LPS encoding regions in the two *L. biflexa* strains maintained separately for 17 years show some evidence of minor accumulation of changes. Comparison of the Paris and Ames strains of *L. biflexa* reveals four indels, three of which are insertions of IS*Lbi1* elements into the coding regions of genes in the Ames strain (LBF_0259, LBF_2295 and LBF_2512). The first two genes encode proteins of unknown function, and the third encodes a protein with a predicted role in lipid metabolism; the significance of these insertions is not known. In the Paris strain, the insertion element is restricted to one copy each on CII and p74; these copies are maintained in the Ames strain. The fourth difference occurs in CII in which an additional 250 bp are found in an intergenic region in the Ames strain. Aside from these differences, the genomes of the Paris and Ames strains are virtually identical; in this article the term *L. biflexa* will refer to the Ames strain unless stated otherwise.

**Figure 2:**
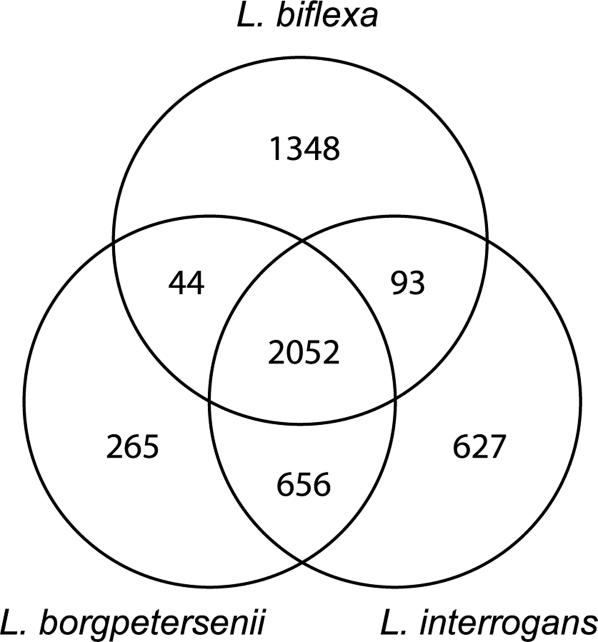
Venn diagram showing numbers of unique and shared genes amongst *L. interrogans*, *L. borgpetersenii* and *L. biflexa.* Orthologous CDS were identified in a pair-wise fashion using Whole-Genome Reciprocal Best-Hit BLAST Analysis [37]. Manual curation ensured a one to one relationship for orthologous CDS, particularly in situations where sets of paralogous CDS existed and in addition evaluated the nature of the relationship between CDS with reciprocal best-hits but low expect values. This analysis was performed using the *L. interrogans* serovar Copenhageni strain Fiocruz, *L. borgpetersenii* serovar Hardjo strain L550 and *L. bifle*xa serovar Patoc strain Ames genome sequences.

Approximately two thirds of the genes in *L. biflexa* have orthologous genes in the pathogens *L. interrogans* and *L. borgpetersenii* (Figure 2), consistent with a common origin for leptospiral saprophytes and pathogens. The genes conserved across the three species are distributed in the two chromosomes of all *Leptospira* species and strains (Table 3). The high sequence identity of the small chromosome *parA* gene product (>87%) between all *Leptospira* spp. suggests a shared ancestry for this replicon. Moreover, there is no difference in inter-chromosomal DNA parameters such as GC%, codon preferences and gene density within the three *L. biflexa* replicons, despite the relatively wide range of these parameters in pathogenic *Leptospira*. By contrast, codon preferences of genes of the leptospiral bacteriophage LE1 and its host, *L. biflexa* [10] are different (data not shown). These data suggest a long-standing relationship between the three *L. biflexa* replicons and perhaps a more recent acquisition of LE1.

**Table 3:** Distribution of the orthologs over the two chromosomes of *Leptospira* spp.

	***L. biflexa*** ** strain Patoc1**	***L. interrogans*** ** strain Fiocuz L1-139**	***L. interrogans strain Lai 56601***	***L. borgpetersenii strain L550***	***L. borgpetersenii strain JB197***
No od CDS shared between C1 replicons (1)	1411 (41.58%)	1429 (39.13%)	1429 (37.41%)	1482 (38.73%)	1448(38.20%)
No of CDS shared between CII replicons (2	80 (28.46%)	83 (27.66%)	83 (26.17%)	82 (24.47%)	82 (25.07%)

(1) Number of CDS (orthologs) found in the large chromosome (CI) of one leptospire that are also found in the large chromosomes of the other four leptospiral large chromosomes. (2) Number of CDS (orthologs) found in the small chromosome (CII) of one leptospire that are also found in the small chromosomes of the other four leptospiral large chromosomes. Putative orthologous relations between two genomes are defined as gene couples satisfying the bi-directional best hit (BBH) criterion or a blastP alignment threshold, a minimum of 40% sequence identity on 80% of the length of the smallest protein. CDS, coding regions.

### The evolution of the *Leptospira* species

Phylogenetic analysis based on comparison of 16S rRNA sequences indicates that members of the family *Leptospiraceae* form the deepest branch in spirochete evolution, with divergence of saprophytic and pathogenic *Leptospira* likely being the result of a single event [13]. Further diversification of species within each of these two evolutionary branches of *Leptospira* is supported by multilocus sequence analysis and DNA renaturation kinetics [3], [14], [15].

**Figure 3:**
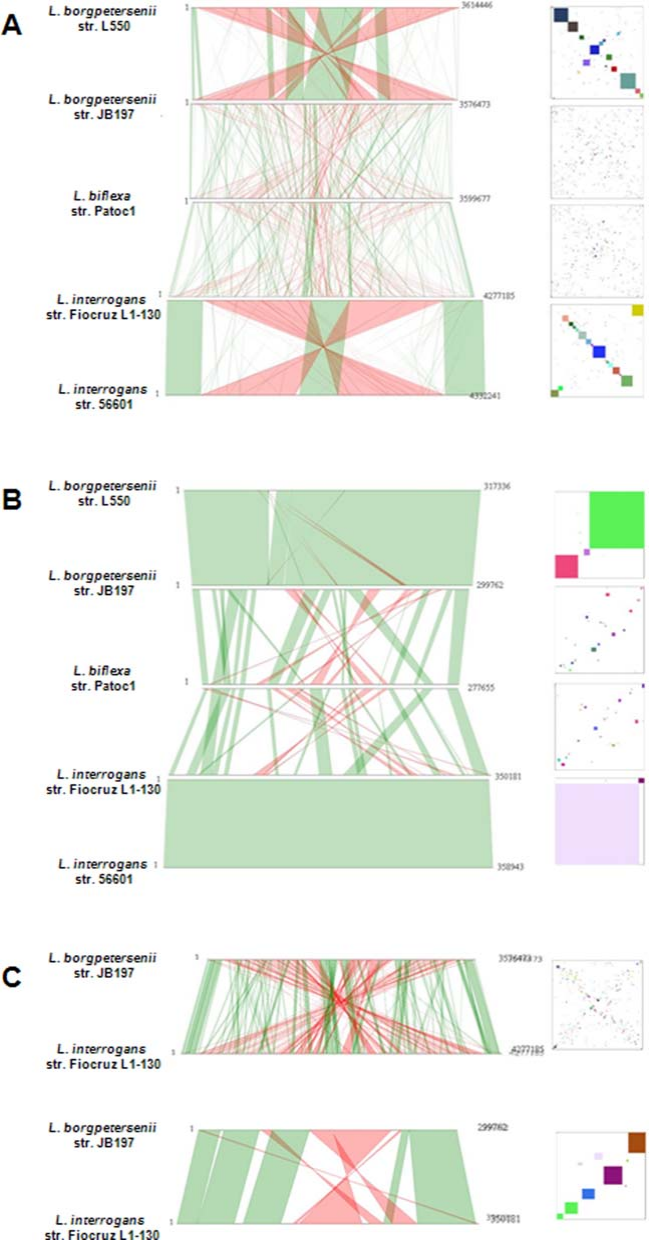
Synteny plot between the five *Leptospira* genomes. The line plots were obtained using synteny results between the large CI(A) or small CII(B) chromosomes of *L. biflexa* serovar Patoc strain Patoc1, *L. interrogans* serovar Lai strain 56601, *L. interrogans* serovar Copenhageni strain Fiocruz L1-130, *L. borgpetersenii* serovar Hardjo strain L550, and *L. borgpetersenii* serovar Hardjo strain JB197. A line plot (C) compares synteny between *L. borgpetersenii* serovar Hardjo strain JB197 and *L. interrogans* serovar Copenhageni strain Fiocruz L1-130. Comparative analysis was performed using the MaGe interface [35] in the SpiroScope database (https://www.genoscope.cns.fr/agc/mage). The minimum size of the synteny groups is set to five genes. In green: synteny groups are organized on the same strand; in red: synteny groups are organized on opposite strands.

We used comparative genome analysis to help identify key features showing patterns of variation consistent with the action of selective evolutionary pressure within *Leptospira*. The *L. biflexa* and *L. borgpetersenii* genomes are similar in size (Table 1), but the gene density in *L. biflexa* is much higher, probably as a result of IS-mediated genome erosion in *L. borgpetersenii*. In contrast, the *L. interrogans* genome is larger, probably reflecting the added genetic information required for survival both within mammalian hosts and aquatic environments, whereas *L. biflexa* and *L. borgpetersenii* are restricted to aquatic and mammalian host environments, respectively. The dearth of IS-elements in the *L. biflexa* genome (five IS-elements) is in stark contrast to their abundance in the *L. interrogans* (36 and 69 IS-elements in the Fiocruz LI-130 and Lai 56601 strains, respectively) and *L. borgpetersenii* (167 IS elements) genomes. The presence of large numbers of IS elements is an indicator of genome plasticity in *Leptospira* species. Taken together, our results suggest that the *L. biflexa* gene order is more likely to have a closer relationship to the progenitor genome for the genus *Leptospira*. There is low synteny between the sequenced pathogenic *Leptospira* (Figure 3) [5], despite the short evolutionary distance separating them.

Gene and functional redundancy is also more common in the pathogens in comparison to the saprophytes (Table 4). The pathogens have more paralogs (excluding transposases), with 203 (5.01%) and 438 (10.52%) paralogs for *L. interrogans* serovar Copenhageni Fiocruz L1-130 and *L. borgpetersenii* serovar Hardjo L550, respectively, compared to 62 paralogs (1.65%) in the saprophyte *L. biflexa*. We identified 43, 67, and 53 loci with gene duplication in the genomes of *L. biflexa*, *L. interrogans*, and *L. borgpetersenii*, respectively. The genome of *L. biflexa* therefore appears to be more stable than those of *L. interrogans* and *L. borgpetersenii*. Moreover, the *L. biflexa* genome (92%) has a greater gene density than the *L. borgpetersenii* (80%) and *L. interrogans* genomes (75%) (Table 1). Greater gene density can contribute to a relatively stable gene order. For example, the spirochete *Borrelia* has a gene density of more than 92% and the *B. garinii* and *B. burgdorferi* genomes are essentially collinear [16].

**Table 4:** Comparative genomics of *Leptospira* spp. Putative orthologous relations between two genomes are defined as gene couples satisfying the bi-directional best hit (BBH) criterion or a blastP alignment threshold, a minimum of 40% sequence identity on 80% of the length of the smallest protein. Putative paralogous relations between two genomes are defined as gene couples satisfying the bi-directional best hit (BBH) criterion or a blastP alignment threshold, a minimum of 60% sequence identity on 80% of the length of the smallest protein.

Paralogs/orthologs	*L biflexa* strain Patoc 1	*L. interrogans* strain Fiocruiz L1-130	*L. borgpetersenii* strain L550	*L. borgpetersenii* strain JB197	*L. interrogans* strain Lai 56601
***L biflexa*** ** strain Patoc 1**	62 (1.5%)	1650 (44.04%)	1635 (43.64%)	1631 (43.53%)	1652 (44.10%
***L. interrogans*** ** strain Fiocruiz L1-130**	1633 (40.35%)	203 (5.01%)	2913 (71.97%)	2907 (71.83%)	3745 (92.53%)
***L. borgpetersenii*** ** strain L550**	1674 (40.23%)	3084 (74.11%)	438 (10.52%)	3936 (94.59%)	3077 (73.94%)
***L. borgpetersenii*** ** strain JB197**	1636 (39.73%)	3052 (74.13%)	3922 (95.26%)	354 (8.59%)	3044 (73.93%)
***L. interrogans*** ** strain Lai 56601**	1638 (39.60%)	3778 (91.34%)	2942 (71.13%)	2934 (70.93%)	204 (4.93%)

### Genetic determinants involved in survival in the environment

The spectrum of ecological niches occupied by diverse *Leptospira* species raises questions as to how the capacity to survive in these diverse environments has evolved within different species in the genus *Leptospira*. We propose that their common progenitor had a genome more like *L. biflexa*. Subsequently, acquisition of genes that enabled *Leptospira* to infect mammals would have expanded the range of environments that it could successfully occupy. Loss of environmental survival genes then would lead to dependence on a mammalian host and eventually return the genome to a smaller size (i.e. *L. borgpetersenii*), consistent with our model for IS-mediated genome degradation [5].

The extensive repertoire of genes (Table 5) encoding proteins involved in signal transduction in *L. biflexa* (287 CDS) compared with *L. interrogans* (214 CDS) and *L. borgpetersenii* (167 CDS) is consistent with an enhanced metabolic capability in *L. biflexa* reflected by its environmental habitat and likely contributes to its enhanced growth rate relative to the pathogens. An analogous situation is seen in *Mycobacterium*, where *M. marinum* which occupies both animal and aquatic environments has many more genes encoding environmental sensing and metabolic proteins than the closely related, obligate human pathogen *M. tuberculosis* [17], [18].

**Table 5:** Distribution of general protein functions between leptospiral species based on the COG function classification scheme.

^♣^COG Function Classification		¥ *L. biflexa*	¥ *L. borgpetersenii*	¥ *L. interrogans*
**INFORMATION STORAGE AND PROCESSING**				
J	Translation, ribosomal structure and biogenesis	154	174	153
K	Transcription	166	104	109
L	Replication, recombination and repair	94	91	102
B	Chromatin structure and dynamics	2	2	2
**CELLULAR PROCESSES AND SIGNALING**				
D	Cell cycle control, cell division, chromosome partitioning	21	22	22
V	Defense mechanisms	39	32	37
T	Signal transduction mechanisms	287	167	214
M	Cell wall/membrane/envelope biogenesis	230	199	218
N	Cell motility	93	84	89
U	Intracellular trafficking, secretion, and vesicular transport	71	73	71
O	Posttranslational modification, protein turnover, chaperones	105	96	100
**METABOLISM**				
C	Energy production and conversion	132	115	119
G	Carbohydrate transport and metabolism	91	76	91
E	Amino acid transport and metabolism	163	136	150
F	Nucleotide transport and metabolism	46	52	52
H	Coenzyme transport and metabolism	119	112	120
I	Lipid transport and metabolism	101	83	99
P	Inorganic ion transport and metabolism	120	72	88
Q	Secondary metabolites biosynthesis, transport and catabolism	35	23	27
**POORLY CHARACTERIZED**				
R	General function prediction only	311	237	294
S	Function unknown	174	157	192
	CDS Not Classified (not related to any COG)	1,266	931	1,245
	Total CDS (count excludes transposases and pseudogenes)	3,590	2,843	3,378

♣ Each COG assignment has been manually curated to ensure consistent classification across orthologous proteins. A feature of the COG scheme is that some COGs have multiple functional classifications.

¥ *L. borgpetersenii* serovar Hardjo strain L550, *L. interrogans* serovar Copenhageni strain Fiocruz, *L. biflexa* serovar Patoc strain Ames

The genomes of *Leptospira* spp. contain a number of genes involved in the production of exopolysaccharides. These genes, such as those encoding glycosyltransferases, alginate biosynthesis, and lipopolysaccharide transport systems, may contribute to colonization of both biotic and abiotic surfaces. When investigating the formation of biofilms on solid surfaces, we have observed the production of a strong biofilm by *L. biflexa* and *L. interrogans* (Unpublished results). The formation of such a biofilm is consistent with the life of saprophytic species in water; it may facilitate *L. biflexa* occupying particular environmental niches. The presence of biofilms may also play an important role in chronic carriage of the pathogen *L. interrogans* in animal reservoirs by facilitating colonization of the renal tubules. Interestingly, genes involved in alginate biosynthesis are present in both *L. biflexa* (11 genes) and *L. interrogans* (8 genes), but are absent in *L. borgpetersenii*, a finding consistent with the reduced environmental survival of *L. borgpetersenii* [5].

### Comparative genomics of pathogenic and saprophytic *Leptospira*

The molecular mechanisms of pathogenesis in leptospirosis remain almost entirely unknown. The set of leptospiral, pathogen-specific genes, defined as those with no orthologous gene in *L. biflexa*, is likely to be enriched for genes that play a role in pathogenesis. Significantly, the majority (893) of these 1,431 genes has no known function (Table S1abc), suggesting the presence of pathogenic mechanisms unique to *Leptospira*. Among the remaining 538 genes, there are several genes encoding response regulators and environment sensing proteins; these genes are likely to represent adaptations that enable survival in environments not encountered by the saprophytic strain. There is an expansion of genes encoding leucine–rich repeat (LLR) proteins from one gene in *L. biflexa* to eight and 18 genes in *L. borgpetersenii* and *L. interrogans*, respectively. Although these LLR proteins have no obvious function, in *Treponema denticola* the LRR protein LrrA appears to have roles in attachment and to, and penetration of, host tissues [19]; the diversity of LRR proteins in pathogenic *Leptospira* may be important for succesful infection of a wide variety of mammalian host species.

The regulation of transcription differs significantly between the saprophytic and pathogenic strains as indicated by the presence of more than 50 saprophyte-specific, and more than 20-pathogen specific, transcription regulators, whereas there are only 27 transcription regulators that are common to all of the sequenced *Leptospira* species.

Because the bacterial surface is an interface between the pathogen and the host, any differences in cell-surface proteins might reflect variation in pathogenesis mechanisms among *Leptospira* spp., which contain a relatively low repertoire of trans-membrane proteins in their outer membranes [20]. However, the outer membranes contain a predominance of lipoproteins, that may be either surface-exposed or located in the periplasm. Predicted lipoproteins are prominent in both saprophytic (164 predicted lipoproteins) and pathogenic *Leptospira* (184 and 130 predicted lipoproteins in *L. interrogans* and *L. borgpetersenii* respectively). Despite these similarities, there are significant differences, most notably the absence in *L. biflexa* of an ortholog of the major outer membrane lipoprotein, LipL32 [20]. Moreover, 89 *L. biflexa* lipoprotein genes have no orthologs in other *Leptospira* species, and more than 90 lipoproteins from the pathogenic species have no orthologs in the *L. biflexa* genome. In addition to LipL32, other characterized lipoproteins that have no orthologs in *L. biflexa* include LipL41, LipL36 and several LipL45 related proteins [20]. However, LipL21, which was reported as a pathogen-specific lipoprotein [21] based on antibody reactivity, has an ortholog in *L. biflexa* with 50% similarity. This level of similarity is consistent with a different function for the LipL21 orthologs in the two *Leptospira* species.

Several putative virulence factors previously identified in pathogenic *Leptospira* spp. are not present in *L. biflexa*, including the Lig surface proteins containing immunoglobulin-like repeats predicted to play a role in the adhesion to host tissues [22]. Similarly, LfhA, a putative factor H binding protein [23] that has also been shown to bind the extracellular matrix protein laminin [24], is shared among pathogenic *Leptospira*, but is lacking in *L. biflexa*. Although the genome of *L. biflexa* contains putative hemolysins [25], its genome is devoid of genes encoding enzymes capable of degrading tissues, such as the range of sphingomyelinases found in pathogenic species [26], [27], [28]. The role of sphingomyelinases in the pathogenesis of leptospirosis has been controversial; are they key virulence factors or do they merely play a role in nutrient acquisition? Their absence in *L. biflexa* strongly supports their involvement in survival within mammalian hosts. Interestingly, the membrane protein, Loa22, the only protein to date that has been shown genetically to be required for virulence in *L. interrogans* [29], has a *L. biflexa* ortholog with 73% similarity. Its role in either pathogenic or saprophytic species is unknown, but its presence in the saprophytic species suggests that it is involved in survival rather than being a direct virulence factor and is consistent with the common progenitor hypothesis.

*L. interrogans* is the most frequently reported agent of human leptospirosis. The disease is also generally more severe with *L. interrogans* than with *L. borgpetersenii* [2]. On this basis we propose that the subset of *L. interrogans* genes that have no orthologs in either *L. biflexa* or *L. borgpetersenii* may contain virulence factors that are responsible for the more severe form of leptospirosis. Other subsets that may be enriched for genes involved in particular biological functions include those genes that have orthologs in *L. biflexa* and *L. interrogans* and not *L. borgpetersenii* which may contain genes involved in survival outside the animal host. While the loss of many genes from the *L. borgpetersenii* genome has occurred through genome reduction [5], the presence of 265 unique genes in *L. borgpetersenii* (Figure 2; Table S1abc) indicates that these genomes have also gained some additional genes during the course of their evolution. The lateral acquisition of genetic material is often associated with IS-elements. While there is an association between IS-elements and genes that are isolate- or species-specific, in particular *in L. interrogans*, no mechanism has been determined. Other possible mechanisms for horizontal gene acquisition may include the involvement of bacteriophage, as integrated prophages or bacteriophage remnants are present.

### The Core Leptospiral Genes

Saprophytic and pathogenic *Leptospira* species belong to two distinct phylogenetic groups, leading us to conclude that the 2,052 genes shared by both groups constitute the core genome of this genus (Figure 2). As expected, many of the functional categories that are involved in essential housekeeping functions, such as DNA and RNA metabolism, protein processing and secretion, cell structure, cellular processes, and energetic and intermediary metabolism, are represented in the core gene set.

The presence of orthologous genes in all the sequenced leptospiral species is an indicator that these genes were acquired prior to the radiation of the genus, and as such, is a strong indicator that these genes have not been laterally acquired. The substantial proportion of the total leptospiral genes that are in this category is perhaps an indicator that lateral transfer of genes into the genus *Leptospira* is a minor contributor to the overall genetic composition of the genus and an indicator that the genus has undergone an extended period of ‘genetic isolation’. This notion is supported by the fact that approximately 20% of the core leptospiral genes are unique to the genus. However, this does not preclude horizontal acquisition of some genes or gene clusters.

### Implications for genetic studies

Despite the development of basic tools such as transposon mutagenesis for the pathogenic species of *Leptospira*, targeted gene inactivation is not yet possible. Therefore, there is likely to be continued interest in the use of *L. biflexa* as a model bacterium for genetic analysis [30]. Knowledge of the distribution of orthologous genes in *L. biflexa* will be an important resource for the elucidation of function for genes common to pathogenic and saprophytic species.

## Materials and Methods

### Sequencing and annotation of the genome of *L. biflexa*

The strain, *L. biflexa* serovar Patoc strain Patoc1, was initially isolated from stream water [31], maintained in the collection of the National Reference Center of *Leptospira* (Institut Pasteur, Paris, France) and designated the Paris strain. A second strain was derived from the same source but kept in the culture collection at the National Animal Disease Center (NADC), Ames, IA since 1990 and is designated the Ames strain. Each strain was colony purified before growth for genomic DNA isolation. For each isolate, genomic DNA was randomly sheared by nebulization (HydroShear, GeneMachines) and the ends repaired enzymatically. Small fragments (∼1.5–4 kb) were ligated either to a derivative of plasmid pGEM7Zf+ (Promega) (Paris strain) or pSMART-HC and pSMART-LC (Lucigen Corp.) (Ames strain). Large (35–45 kb) DNA fragments were ligated to fosmid pCC1FOS (Epicentre, Madison, WI) (Paris strain). Intermediate sized DNA fragments (7–12 kb) were prepared from partially *Bam*HI-digested Ames strain DNA and ligated into pZERO-1 (Invitrogen). Plasmid DNA preparation was performed with the TempliPhi DNA sequencing template amplification kit (GE Healthcare -Bio-Sciences) or SprintPrep plasmid preparation kits (Agencourt Bioscience). Fosmid DNA purification was performed with the Montage BAC Miniprep96 Kit (Millipore) [25]. Sequencing reactions were performed from both ends of DNA templates using ABI PRISM BigDye Terminator cycle sequencing ready reactions kits and run on a 3700 or a 3730 xl Genetic Analyzer (Applied Biosystems) at the Genomics platform (Pasteur Genopole Île-de-France), the Australian Genome Research Facility, Brisbane, Australia or at the Genomics Facility at the NADC. Base-calls from sequence data were made using Phred [32]. Sequences not meeting our production quality criteria (at least 100 bases called with a quality over 20) were discarded. The traces were assembled using Phrap and Consed [33].

Whole genome shotgun sequencing was performed until approximately 6× genome coverage was achieved. Autofinish [34] was used to design primers for improving regions of low quality sequence and for primer walking along templates that spanned the gaps between contigs. Several strategies were used to orientate contigs to enable directed PCR-based approaches to span gaps between contigs. These strategies included Blast-based approaches that identified contig ends with hits to the same gene or to genes within the same locus, provided that there was conservation of gene order in both the *L. interrogans* and the *L. borgpertersenii* genomes. Repeated sequences longer than 600 bases, such as IS-elements and the *rrl* and *rrn* genes, were identified and curated manually to ensure that at least two templates spanned these regions, thus confirming the assembly of these regions. At this stage, single, circularised contigs representing CII and and p74 were identified. Combinational PCR was used to close the gaps between the final contigs making up CI. Outward-directed primers were designed for each of the contig ends; the primer sequences were subsequently checked and confirmed to be unique on the genome. The combinational PCR process required approximately 600 PCR reactions pairing each of the primers. In each instance where a gap was spanned, the size of the PCR product was less than 2 kb. The sequence of these PCR products was determined and added to the assembly to enable the closing of CI. PCR with independent primers was used to confirm the joins determined by the combinational PCR. In addition, for the Paris strain five fosmid clones were completely sequenced by transposon-assisted sequencing (Finnzymes, TGS II kit). Seven fosmid clones were also selected by Southern hybridization and sequenced in order to verify the 23S RNA and 16S RNA regions and 4 repeat regions. Validation of the final assembly was achieved by comparison of *in silico* digestion patterns with macrorestriction patterns obtained by PFGE with *Not*I and *Asc*I. For each genome, the error rate was less than 1 error per 10,000 bp.

The complete genome sequence was obtained from 58,663 and 40,260 sequences for the Paris and Ames strains respectively (giving >8× and 7× coverage). For the Paris strain, coding sequences (CDSs) likely to encode proteins were predicted with the AMIGene software [35]. Annotation was performed as described previously [25] using the MaGe annotation platform [36]. All the data were stored in SpiroScope, a relational database which is publicly available (http://www.genoscope.cns.fr/agc/mage). The Ames strain was annotated as described previously for the *L. borgpetersenii* serovar Hardjo genome using the Wasabi interactive platform [5].

## Supporting Information

Figure S1 (0.05 MB DOC)

Table S1 Supplementary Table 1 a, b and c
(0.46 MB XLS)
