# Supplementary material for: Genome Sequence of the Saprophyte Leptospira biflexa Provides Insights into the Evolution of Leptospira and the Pathogenesis of Leptospirosis
Source: PLoS One. 2008 Feb 13;3(2):e1607. doi: 10.1371/journal.pone.0001607 (PMC2229662; doi:10.1371/journal.pone.0001607)
Supplement: Supplementary file 1 [file pone.0001607.s001.doc]

Figure S1

A

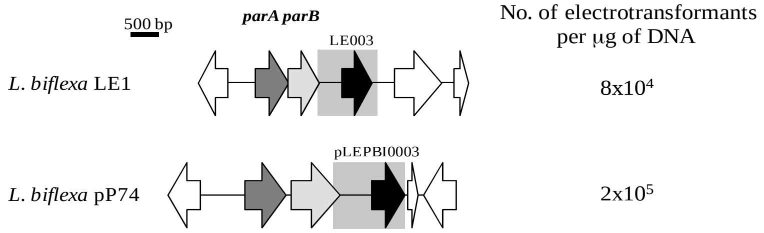

B

```
               . . . .10 . . . .20 . . . .30 . . . .40 . . . .50 . . . .60 . . . .70 . . . .80 . . . .90 . .
RepLE1     ...MRTGLWIPVEIEVLPLNLTEKVLLAEIVSLDRAGECFASNEHFSKLLGVRSDSVSRIISKLKKVGLVKQTGFDGRRRKLIPILESKSQIKQE
ReppP74    MKTNRKGIWIPVWIENLNLSHSQTKLFAEIVSLYENGGCFASNRYFSEILGLKADTISRLITSLKQLGILEQTGFDGRRRFLKPILQFQPEALEK

           . 100 . . . 110 . . . 120 . . . 130 . . . 140 . . . 150 . . . 160 . . . 170 . . . 180 . . . 190
RepLE1     .PILSPEPKQAVPKTKSRIGMSAEAAFAISNVPLKRVQLQN..NVQKSWDEFLNWSRGRVAPTTWDLISKVSRPEDLKGSASLMWKNWAIL....
ReppP74    NPSLTN..VTISNKIQTTTANNSKPALDSSYVPSITVQLKNTVHTKTSFEEFKVWSESTLSHSTHSKILHFTSPDSMEESLQRIWNQWMEKTKPK

            . . . .
RepLE1     .........
ReppP74    SNSFQVGIV
```

Identification of the replication origin of p74.

A. Cloning a 1.4-kb DNA fragment of p74 into the kanamycin-resistant plasmid pGKm, which is derived from pGEM-7Zf(+) (Promega, Madison, WI), enables autonomous replication of the vector in *L. biflexa*, as previously shown for the LE1 replication origin (Saint Girons et al., 2000). The minimal replication region is shaded in grey. The putative *parA* and *parB* genes are also indicated. *L. biflexa* resistant colonies appeared at a mean frequency of 2.6 x 10^5 transformants per µg of DNA. *Leptospira* cells were electrotransformed as previously described (Saint Girons et al., 2000). The authenticity of transformants was established by preparing minipreps from *L. biflexa* resistant transformants, using them to transform *E. coli* and then isolating plasmid DNA from the resultant transformants and confirming their restriction profiles (data not shown).

B. Sequence alignment of pLEPBI003 (Repp74) and LE003 (RepLE1) proteins. Conserved residues conserved are shaded.
